# Epigenetic Regulation of Enteric Neurotransmission by Gut Bacteria

**DOI:** 10.3389/fncel.2015.00503

**Published:** 2016-01-08

**Authors:** Tor C. Savidge

**Affiliations:** ^1^Department of Pathology and Immunology, Baylor College of MedicineHouston, TX, USA; ^2^Texas Children's Microbiome Center, Texas Children's Children HospitalHouston, TX, USA

**Keywords:** microbiome, metabolome, neurotransmitters, nitric oxide, epigenetics, neuropathy, intestinal disease, enteric nervous system

## Abstract

The Human Microbiome Project defined microbial community interactions with the human host, and provided important molecular insight into how epigenetic factors can influence intestinal ecosystems. Given physiological context, changes in gut microbial community structure are increasingly found to associate with alterations in enteric neurotransmission and disease. At present, it is not known whether shifts in microbial community dynamics represent cause or consequence of disease pathogenesis. The discovery of bacterial-derived neurotransmitters suggests further studies are needed to establish their role in enteric neuropathy. This mini-review highlights recent advances in bacterial communications to the autonomic nervous system and discusses emerging epigenetic data showing that diet, probiotic and antibiotic use may regulate enteric neurotransmission through modulation of microbial communities. A particular emphasis is placed on bacterial metabolite regulation of enteric nervous system function in the intestine.

## Introduction

The intestine has evolved efficient digestive, endocrine and immune functions in concert with its microbiota—a vast and complex symbiotic ecosystem comprising bacteria, viruses, fungi, protozoa, and archaea living in close proximity to the host (Bäckhed et al., 2005; Jumpstart Consortium Human Microbiome Project Data Generation Working Group, 2012; Yatsunenko et al., 2012). Working in synergy with these microorganisms, the gut-microbiota axis provides the host with adequate nutrients and energy for good health, growth and reproduction. While little is known about the virome and eukaryotic microorganisms in the gut, the human intestinal microbiota is comprised of over 50 different bacterial phyla that are dominated by Bacteroidetes, Firmicutes, and Actinobacteria in healthy adults (Bäckhed et al., 2005; Jumpstart Consortium Human Microbiome Project Data Generation Working Group, 2012; Yatsunenko et al., 2012). Age is a significant determining factor in microbiota composition, as is genetic hardwiring and host immune responses that help to shape our gut microbial communities (Yatsunenko et al., 2012; Kelder et al., 2014). Diet, probiotic, and antibiotic use also modulate intestinal microbiota composition and represent some of the best studied examples of how epigenetic signals can potentially cause permanent alterations to microbial community structure and function in humans (Turnbaugh et al., 2006, 2008; Yatsunenko et al., 2012; Hemarajata and Versalovic, 2013).

Epigenetics is defined as heritable changes in gene expression that occur without coding changes in DNA sequence and often involve posttranscriptional and/or posttranslational signals from the environment. It is increasingly appreciated that the gut microbiota can contribute to human disease, not only as infectious agents but also by altering exposure to dietary, pharmacological and environmental factors that may constitute a disease risk (Turnbaugh et al., 2006, 2008; Hemarajata and Versalovic, 2013). Short chain fatty acids represent one of the best studied epigenetic examples of how microbial metabolites can modulate host function (Choi and Friso, 2010; Donohoe et al., 2011). Short chain fatty acid producing bacteria are especially sensitive to antibiotics and antimicrobial therapy may directly influence epigenetics by inhibiting histone deacetylase activity, DNA methylation status and gene transcription in the host. Tissues immediately exposed to such microbial metabolites are most at risk of aberrant gene regulation and include the enteric nervous and immune systems, as well as dysregulation of intestinal epithelial stem cells that may lead to neoplasia (Berni Canani et al., 2012). Because microbial drug and dietary metabolites, including bacterial and viral immunomodulators of toll-like pattern recognition receptors, serve as epigenetic activators of host gene expression this is an area of intense investigation that requires a better understanding of microbial community dynamics and altered biochemical function associated with disease risk in humans.

Massive parallel sequencing of bacterial genes has transformed our understanding of gut microbial community dynamics in intestinal disease progression, where there is often an inferred shift in bacterial taxa and function (Jumpstart Consortium Human Microbiome Project Data Generation Working Group, 2012; Yatsunenko et al., 2012). Although, the use of next generation sequencing technology has significantly advanced our knowledge of the intestinal microbiome, it is also fraught with limitations (Polz and Cavanaugh, 1998; Schloss et al., 2011; Edgar, 2013). Bacterial ribosomal 16S RNA profiling is inherently biased due to estimation of microbial diversity being reliant upon a gene that varies in copy number and sequence per microbe. Moreover, nucleic acid amplification and software algorithms used for community structure analysis introduce marked biases relative to the variable 16S rDNA gene region being studied. The short DNA sequence reads generated also make bacterial species identification difficult. Whole shotgun genome sequencing offers certain advantages over 16S rDNA profiling, but this technology is low throughput, expensive and time consuming by comparison. Our own experiences strongly support independent validation of species of interest using complementary quantitative methods, although identification of disease-associated microbes can prove problematic for further study since most gut bacteria are difficult to isolate, culture and adapt for *in vivo* testing. Nevertheless, these systems biology approaches have provided important insights into how the gut-microbiota axis may constitute a significant risk factor in diverse human pathologies, including infection, obesity, cancer, diabetes, autism, autoimmune, irritable, and inflammatory bowel diseases (this list is by no means exhaustive; Sharkey and Savidge, 2014).

Given that a bacterial presence in the intestine exerts major host physiological responses, it seems highly likely that some of these effects involve signaling to and from the enteric nervous system. The enteric nervous system controls virtually all known gut functions and has evolved sophisticated regulatory circuits to manage clinical risk factors that contribute to the disease susceptibilities listed above (Lomax et al., 2010; Furness, 2012; Sharkey and Savidge, 2014). Whether, gut microbes directly modulate these systems of checks and balances is not clear and is a focus of this mini-review.

## Nervous system control of intestinal function

**Figure 1:**
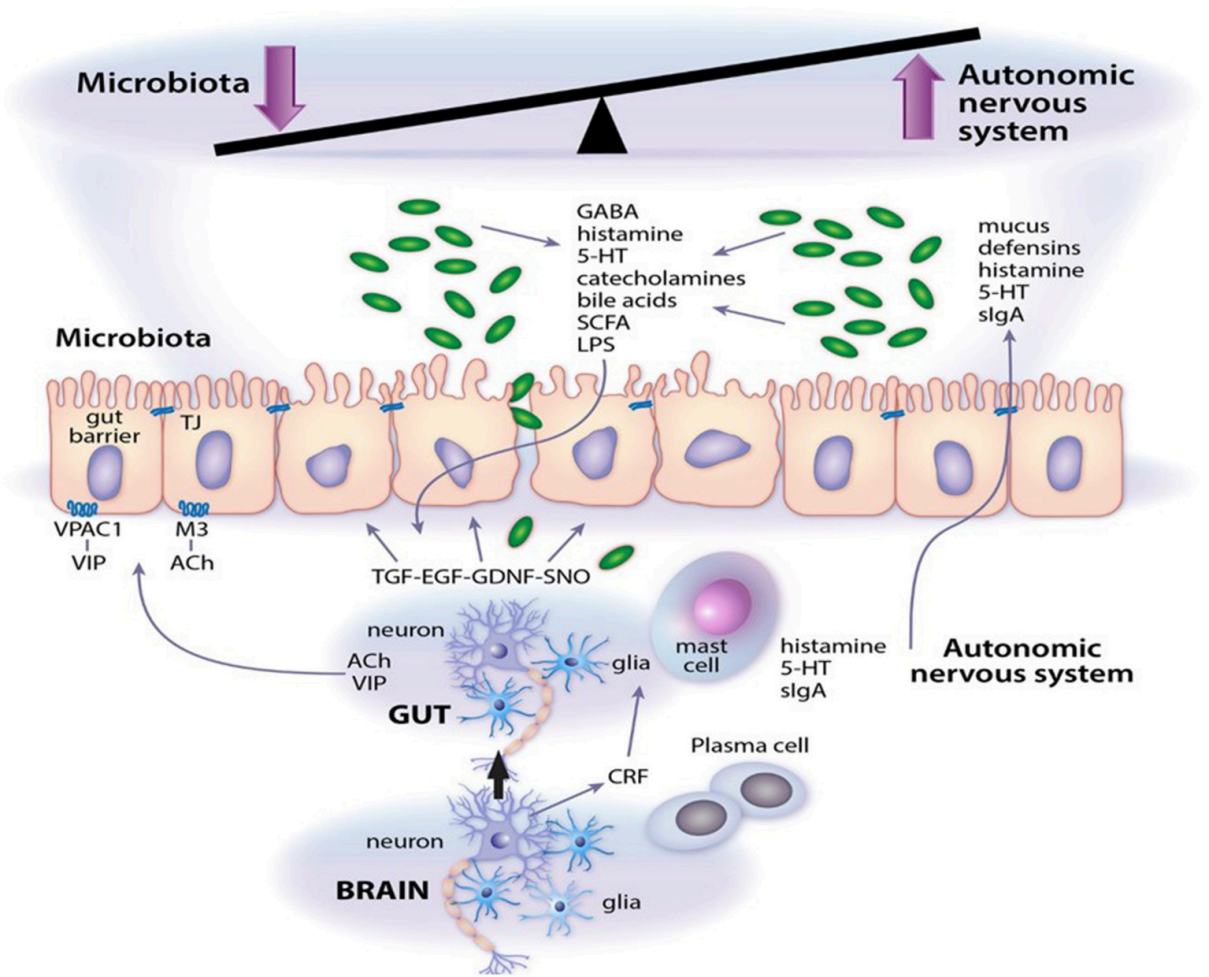
**Microbial neurotransmitter crosstalk with the autonomic nervous system**. As outlined in the article, a system of checks and balances operate to regulate gut function. Abbreviations: 5-HT, serotonin; Ach, acetylcholine; CRF, corticotrophin releasing factor; EGF, epidermal growth factor; GDNF, glial cell line-derived neurotrophic factor; LPS, lipopolysaccharide; M3, M3 muscarinic receptor; SCFA, short chain fatty acids; sIgA, secretory IgA; SNO, s-nitrosothiol; TGF, transforming growth factor; VIP, vasoactive intestinal peptide; VPAC1, VIP and PACAP receptor 1.

Enteric neuropathy and aberrant neurotransmission are common findings in a range of intestinal diseases where significant shifts in microbial community dynamics are also apparent. A few well defined and/or extensively investigated examples include opportunistic infection by enteric pathogens such as *Clostridium difficile* (Savidge et al., 2011); intestinal barrier failure and sensitization to food antigens (Devkota and Chang, 2013); colorectal cancer; imbalance in humoral and cell-mediated immunity in inflammatory bowel diseases and necrotizing enterocolitis (Margolis and Gershon, 2009; Lomax et al., 2010; Knights et al., 2013); constipation and adverse stress signals emanating from the central nervous system that contribute to functional abdominal pain (Chen et al., 2003; Lyte et al., 2011; Simrén et al., 2013). Disease pathogenesis may initiate at several levels in the intestine, all of which fall under the umbrella control of the autonomic nervous system (which includes the enteric nervous system). The gut also receives functional signals from central spinal, vagal, and sacral afferent terminals, although their respective contributions vary in different regions of the gastrointestinal tract (Lomax et al., 2010; Furness, 2012; Sharkey and Savidge, 2014). The enteric nervous system consists primarily of intrinsic primary afferent, motor and interneurons that are arranged in ganglionated plexi (Sharkey and Savidge, 2014; Lomax et al., 2010; Furness, 2012). It is also innervated by terminals from extrinsic primary afferents, parasympathetic and sympathetic nerves. Neuronal control of gut function is mediated by muscarinic cholinergic, vasoactive intestinal peptide, and nitric oxide signaling pathways (Savidge, 2011) that relay their communications through intermediary cell types, e.g., enteric glia (Gulbransen and Sharkey, 2012), Interstitial cells of Cajal (Farrugia and Szurszewski, 2014), epithelial and smooth muscle cells (Sharkey and Savidge, 2014), immune effectors cells (Wang and Kasper, 2014) that initiate their own physiological signals. Enteric glia are especially interesting cellular targets in the enteric nervous system since these are highly responsive to microbial, luminal and inflammatory signals that regulate intestinal barrier function, immune responses, secretion and motility (Savidge et al., 2007; Gulbransen and Sharkey, 2012; Sharkey and Savidge, 2014; Kabouridis et al., 2015; MacEachern et al., 2015). Moreover, using elegant targeted transgenic technology enteric glia are emerging as active regulators of neurotransmission and neuroplasticity in the intestine (*see chapter by Gulbransen*). It is not clear whether enteric glia in mucosal sites also regulate enteric nervous system function, but this glial population is especially responsive to microbial signals (Kabouridis et al., 2015) and is ideally positioned to mediate signals from the gut lumen to neuronal primary afferents and to neuronal plexi in submucosa and muscularis layers (Savidge et al., 2007; MacEachern et al., 2015). The key points of this mini-review are highlighted in Figure 1, which also consider microbial-derived neurotransmitters as emerging effector signals of the enteric nervous system.

## Excitatory signals along the gut-microbiota axis

Several studies have demonstrated profound microbial influences on enteric and central nervous systems, and *visa versa* (Collins et al., 2012; Dinan and Cryan, 2012; Bienenstock et al., 2015). Notable findings include microbiota-induced excitability of after-hyperpolarization (AH) intrinsic primary afferent neurons in the intestine, which was not observed in germfree animals lacking gut bacteria (McVey Neufeld et al., 2013). Intrinsic primary afferent neurons also show increased membrane polarization and input resistance in germfree mice, and colonization with a defined bacterial community restores normal neuronal excitability. Emerging data from our and collaborator groups show that similar changes in excitability can be elicited by luminal application of microbial metabolites and toxins (Dann et al., 2015; Koussoulas et al., 2015). Sensitized responses are also evident peripherally in skin nerve recordings indicating that microbial signals do not only act locally in the intestine. Recent work indicates that calbindin-immunoreactive neurons in the myenteric plexus may be involved in mediating these microbiota-regulated signals (McVey Neufeld et al., 2015), but it is still not clear which bacteria activate these neurons and how microbial signals are relayed to the myenteric plexus since activation may involve orchestration of signals from local non-neuronal cells. Recent evidence suggests that action potential transmission could relay such luminal-myenteric signals (Mao et al., 2013), as well as nitric oxide intermediate signals which can relay mucosal secretory signals in the opposite direction (Savidge et al., 2007; MacEachern et al., 2015).

Recent reports identified spore-forming gut bacteria as potential members of the microbiota community that elicit neuronal excitability after signaling to enteroendocrine cells in the colon (Reigstad et al., 2015; Yano et al., 2015). Short chain fatty acids, vitamin E derived anti-oxidants and bile acids were shown to promote serotonin synthesis in enteroendrocrine cells (but not in enteric neurons) by inducing tryptophan hydroxylase-1 gene expression, the major rate limiting enzyme in the conversion of dietary tryptophan to serotonin. These host-microbiota interactions significantly impact both local intestinal (e.g., motility) and systemic functions (e.g., via increased carriage of serotonin by platelets). However, it remains to be established whether enteroendocrine cells serve as universal epithelial intermediates in gut-microbiota axis signaling since microbial-derived factors can also exert direct effects on enteric neurons and glia (Sharkey and Savidge, 2014; Kabouridis et al., 2015). In particular, enteric glia are highly responsive to luminal microbial signals and as such may serve as important regulators of both enteric nervous system and mucosal homeostasis (Savidge et al., 2007; Gulbransen and Sharkey, 2012; Kabouridis et al., 2015; MacEachern et al., 2015). In another recent study, microbial lipopolysaccharide and other products operating via an alternative mechanism were shown to cross-signal between enteric neurons and closely juxtaposed muscularis macrophages (Muller et al., 2014). Microbial-induced secretion of colony stimulating factor-1 by enteric neurons triggered activation of muscularis macrophages and release of bone morphogenetic protein 2, which modulated intestinal motility (via an unknown mechanism).

All of these microbial signals appear to significantly impact intestinal physiology by modulating motility and immune function. It remains to be determined whether similar metabolites also activate communications to the central nervous system along the gut-brain axis. Furthermore, because gut bacteria in mice are generally not good community models for the human intestinal microbiota (Nguyen et al., 2015), it is not clear whether similar interactions are also evident in the human enteric nervous system.

It is interesting to note that the converse signals also exist. The host is able to specifically communicate with spore forming bacteria in the intestine using bioactive metabolites, for example via the recently characterized CspC bile acid germinant receptor on *C. difficile* spores (Francis et al., 2013). Certain primary bile acids are able to promote spore germination (cholate), whereas others (chenodeoxycholate) inhibit this receptor and prevent bacterial growth (Sorg and Sonenshein, 2009). Bile acids are therefore able to exert diverse regulatory signals that target both microbe and host cells, e.g., by activating bile acid G-protein coupled receptors (TGR 5) on intrinsic primary afferent neurons (Alemi et al., 2013). Another, example of how the host may communicate with gut bacteria is provided by norepinephrine, the main catecholamine neurotransmitter used by the sympathetic nervous system (Chen et al., 2003; Freestone et al., 2008; Hughes et al., 2009; Lyte et al., 2011). Norepinephrine also serves as a potent quorum sensing signal (auto-inducer) in bacteria, such as *Escherichia coli*. Quorum sensing is a cell-to-cell signaling mechanism used by bacteria to communicate with each other by responding to hormone auto-inducers. In this regard, the host nervous system may directly communicate with microbial communities to regulate bacterial growth, biofilm formation and virulence mechanisms, including toxin production in the intestine. Enterohemorrhagic *E. coli* and *Campylobacter jejuni* are examples where virulence is regulated by catecholamines secreted either by the host or by gut microbes themselves modulate gut function, although how neurotransmitters released by the autonomous nervous system reach the intestinal lumen in an active form remains to be characterized. It remains to be determined how bacterial signals fit into the hierarchical regulation of intestinal physiology, but it is apparent that microbial signals must be considered as essential elements in enteric nervous system control of gut function and host defense.

## Epigenetic regulators of microbial neurotransmitter signals

An emerging literature now supports the idea that normal intrinsic and extrinsic neurotransmission in the enteric nervous system is governed, in part, by bacterial-derived metabolites. However, with the exception of *Clostridium difficile* infection where fecal microbiota transplantation and restoration of a functional intestinal ecosystem has unequivocally demonstrated an unparalleled clinical benefit (Centers for Disease Control and Prevention (CDC), 2013; Sharkey and Savidge, 2014), a direct beneficial or pathogenic role for gut microbes in other human enteric diseases is less certain. Minor alterations in food preferences, intestinal motility, host immune function, prescribed drug or probiotic use in the sick can rapidly alter microbial community dynamics in a misleading manner that is suggestive of a disease association or benefit. Carefully, controlled prospective clinical studies utilizing state-of-the art longitudinal systems biology analysis are needed to address these important issues, especially in view that maternal epigenetic signals may already be exerting developmental disease susceptibility in the young (Lewis et al., 2015).

**Figure 2:**
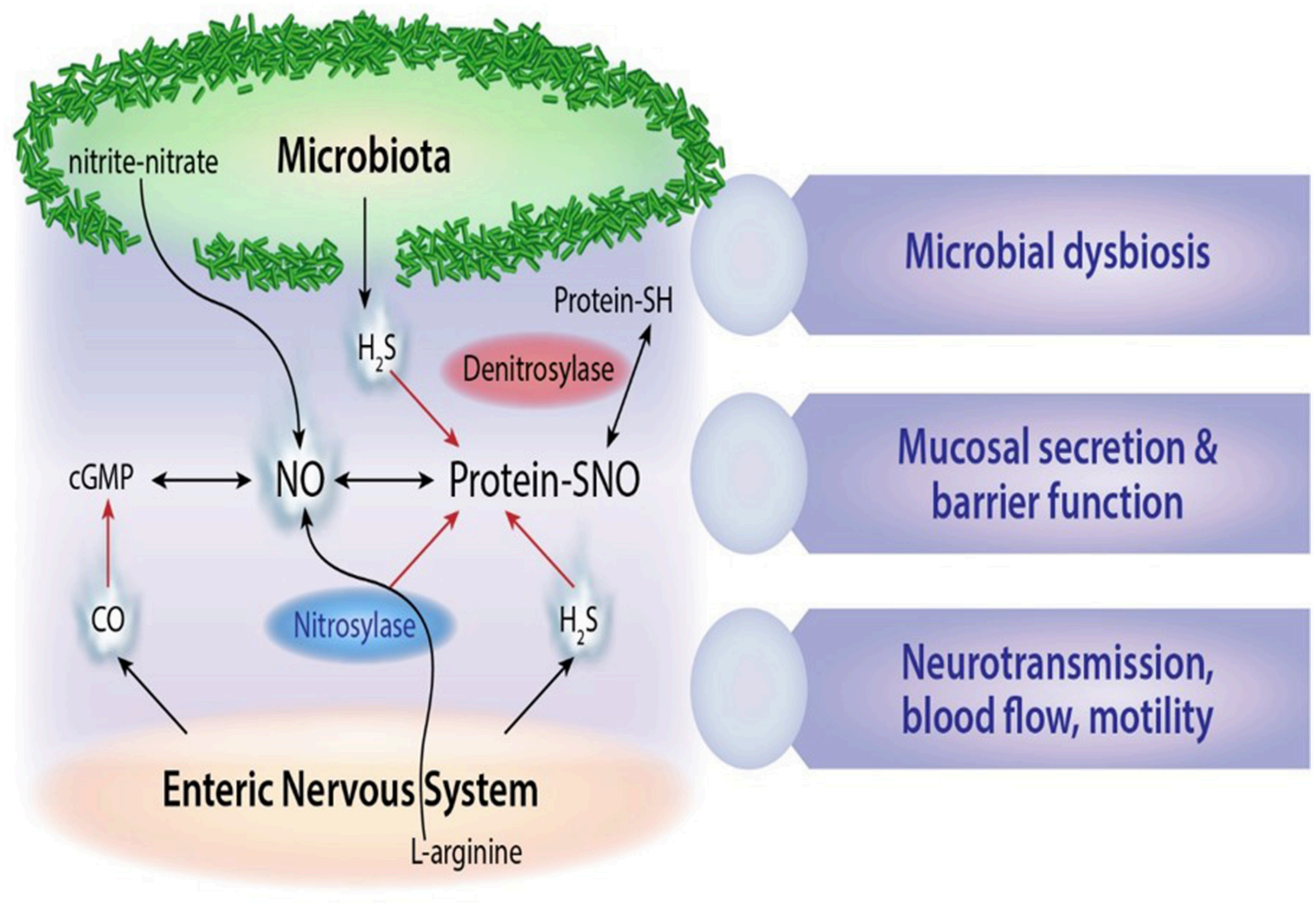
**Microbial gaseous neurotransmitters**. Schematic outline of microbial derived nitric oxide (NO), S-nitrosothiol derivatives (SNO), and hydrogen sulfide (H_2_S) signals and their cross-interactions with carbon monoxide (CO) neurotransmitters in the enteric nervous system.

Altered metabolic activity of gut microbes has been implicated in aberrant regulation of enteric nervous system function, and in some cases clinical symptoms may be alleviated by altering microbial communities using prebiotics, probiotics and antibiotics (Hemarajata and Versalovic, 2013; Koussoulas et al., 2015). Epigenetic influences and early life stressors that adversely affect intestinal microbiota development may have long-term metabolic and immune consequences for human health. Notable examples include regulation of extreme nutritional status in obesity (Ridaura et al., 2013) and kwashiorkor disease (Smith et al., 2013), or in antibiotic-associated disease pathogenesis resulting from the expansion and translocation of resistant gut bacteria e.g., vancomycin-resistant enterococcus (Garbutt et al., 1999). High fat diets and foods enriched in biogenic amino acids such as L-glutamate are also known to have profound effects on both intestinal microbiota composition and nervous system function. Furthermore, the intestine is a rich source of nitric oxide and hydrogen sulfide neurotransmitters generated from bacterial conversion of dietary nitrites and nitrates (Milkowski et al., 2010), as is shown in Figure 2. *E. coli* is a prominent bacterial species that generates bioactive nitric oxide in both the small and large intestine using different oxygen dependent mechanisms (Seth et al., 2012). Other nitrate-reducing bacteria include *Veillonella* and *Actinomyses* spp which are active nitric oxide producers in entero-salivary secretions (Hyde et al., 2014).

Antibiotic use during early life is also reported to have lasting consequences on host physiology by altering intestinal microbiota composition and function (Cox et al., 2014; Lewis et al., 2015). This finding has been exploited for decades by the agricultural industry to promote weight gain in livestock. Potentially, harmful consequences for human health are only now being realized. For example, primary disturbances in microbiota composition by low dose penicillin, a β-lactam antibiotic class most commonly prescribed to young children, promotes excessive adiposity in adulthood that is distinct from diet or genetic-induced obesity (Cox et al., 2014). Several protective bacterial taxa, including Lactobacillus, were reportedly implicated by modulating host metabolism in a manner that may alter enteric nervous system function. This builds on earlier reports that enteric microbes excite intrinsic primary afferent neurons. For example, *Bacteroides fragilis* induces excitability in myenteric AH neurons via signals that are linked to the presence of polysaccharide A in its outer membrane wall (Perez-Burgos et al., 2015). A preliminary study suggests that signaling associated with this probiotic may confer health benefit to gastrointestinal symptoms and behavior in individuals with autism spectrum disorders. Similarly, *Lactobacillus reuteri* is a probiotic bacterium that enhances colonic afferent excitability, in part through modulation of TRPV1 (Perez-Burgos et al., 2015) and calcium-activated potassium channels in AH neurons (Kunze et al., 2009). Although the active metabolite(s) from *Lactobacillus reuteri* has not been clearly characterized, this microorganism is a potent producer of histamine; an important neurotransmitter associated with pathology in the intestine where it regulates immune function, motility, permeability, and secretion (Hemarajata and Versalovic, 2013). Naturally, antibiotic resistant Lactobacilliaceae in patients may therefore be associated with elevated histamine signaling. Similarly, depletion of short chain fatty acid producing bacteria in patients receiving broad spectrum antibiotics may exert epigenetic signals on enteric neurons and glia that result in altered immune responses, intestinal motility and luminal pH. Bacterial signals also target enteric vagal and spinal innervation, although excitability appears to be more species restricted (Bienenstock et al., 2015).

Environmental stressors also impact the host's ability to regulate intestinal physiology, e.g., immune surveillance of the gut microbiota is modulated by autonomic nervous system control of bacterial translocation from the colon. Although an expansive literature demonstrated stress-induced alterations to intestinal permeability and related activation of mast, neuronal and glial cells in the gut wall (Lomax et al., 2010; Collins et al., 2012; Dinan and Cryan, 2012; Furness, 2012; Hemarajata and Versalovic, 2013; McVey Neufeld et al., 2013; Simrén et al., 2013; Sharkey and Savidge, 2014; Bienenstock et al., 2015), less is known about the direct effects of stress on intestinal microbial function. Distinct stress-related outcomes can be associated with central versus enteric signals to the intestinal microbiota. For example, cold stress in humans induces mast cells to release histamine in the small intestine (Santos et al., 1998); whereas central nervous system administration of a thyrotropin releasing hormone analog—a central effector to cold stress—induces serotonin release into the gastric lumen (Stephens and Tache, 1989). Environmental stressors also act on gut-microbiota interactions via the hypothalamic pituitary adrenal axis which is a known regulator of gut microbiota composition and function. Thus, altered microbiota composition and diversity associated with dietary, environmental or psychological stressors may contribute directly to intestinal-related disease, and subsequently may modulate autonomic output by changing the brain's response to environmental and internal stimuli, for example via intestinal synaptic signaling that interconnects primary intrinsic afferents with the vagus nerve (Perez-Burgos et al., 2015). This may potentially lead to a maladaptive cycle of psychological and psychiatric disorders that are often associated with intestinal disease e.g., autism spectrum disorders and Parkinson's disease. It is interesting to note that gut microbiota-derived metabolites are now also implicated in the development and function of central and other peripheral nervous systems, but these observations fall beyond the scope of this mini-review.

## Conclusions

An emerging concept in intestinal disease associated with enteric neuropathy is that microbiota signals can mediate some of the effects, whether beneficial or detrimental. Preliminary studies implicate the enteric nervous system as an important regulatory signal of intestinal microbial community structure and function. Microbial communities themselves are prone to epigenetic signals that are associated with disease and may contribute to the disease pathogenesis by eliciting neurotransmitter profiles that interfere with enteric nervous system homeostasis. Microbial-derived neurotransmitters include regulators such as serotonin, histamine and catecholamines that operate in a coordinated manner with bacterial gaseous transmitters—hydrogen sulfide and nitric oxide—which are being independently pursued as intestinal therapeutics (Savidge, 2014). Careful examination of cross-regulatory circuits between the enteric nervous system and microbial neurotransmission needs to be conducted to assess whether a combinatorial therapeutic approach may prove more effective in enteric neuropathy. Manipulation of the gut microbiota offers a relatively unexplored strategy for therapeutically targeting intestinal disease and will likely generate an extensive repertoire of druggable targets that do not interfere with host nervous systems. Future work needs to characterize the specific bacterial neurotransmitters that synergize or counterbalance signals emanating from autonomic and central nervous systems.

## Author contributions

TS wrote the manuscript.

### Conflict of interest statement

The author declares that the research was conducted in the absence of any commercial or financial relationships that could be construed as a potential conflict of interest.
